# Bifurcated Topological Optimization for IVIM

**DOI:** 10.3389/fnins.2021.779025

**Published:** 2021-12-15

**Authors:** Shreyas Fadnavis, Stefan Endres, Qiuting Wen, Yu-Chien Wu, Hu Cheng, Serge Koudoro, Swati Rane, Ariel Rokem, Eleftherios Garyfallidis

**Affiliations:** ^1^Intelligent Systems Engineering, Indiana University, Bloomington, IN, United States; ^2^Faculty of Production Engineering, Leibniz Institute of Materials Engineering (IWT), Bremen, Germany; ^3^Department of Chemical Engineering, Institute of Applied Materials, University of Pretoria, Pretoria, South Africa; ^4^Radiology & Imaging Sciences, Indiana University School of Medicine, Indianapolis, IN, United States; ^5^Psychological and Brain Sciences, Indiana University, Bloomington, IN, United States; ^6^Department of Radiology, University of Washington, Seattle, WA, United States; ^7^Department of Psychology and eScience Institute, University of Washington, Seattle, WA, United States

**Keywords:** simplicial homology, diffusion MRI, global optimization, separable non-linear least squares, variable projection, diffusion microstructure, intravoxel incoherent motion

## Abstract

In this work, we shed light on the issue of estimating Intravoxel Incoherent Motion (IVIM) for diffusion and perfusion estimation by characterizing the objective function using simplicial homology tools. We provide a robust solution via topological optimization of this model so that the estimates are more reliable and accurate. Estimating the tissue microstructure from diffusion MRI is in itself an ill-posed and a non-linear inverse problem. Using variable projection functional (VarPro) to fit the standard bi-exponential IVIM model we perform the optimization using simplicial homology based global optimization to better understand the topology of objective function surface. We theoretically show how the proposed methodology can recover the model parameters more accurately and consistently by casting it in a reduced subspace given by VarPro. Additionally we demonstrate that the IVIM model parameters cannot be accurately reconstructed using conventional numerical optimization methods due to the presence of infinite solutions in subspaces. The proposed method helps uncover multiple global minima by analyzing the local geometry of the model enabling the generation of reliable estimates of model parameters.

## 1. Introduction

Quantifying tissue microstructure with model-based analysis of diffusion MRI relies on an ill-posed inverse problem of fitting non-linear biophysical models (Tarantola, 1998; Jelescu et al., 2016; Novikov et al., 2018b). From this realm of microstructure models, we focus on estimating the parameters of Intravoxel Incoherent Motion (IVIM) that aims at disentangling diffusion signal contributions from two different stochastic processes (diffusion and perfusion) (Novikov et al., 2014, 2018a; Nedjati-Gilani and Alexander, 2015). The IVIM model uses data collected with the Pulsed Gradient Spin Echo Sequence (PGSE), regularly used to measure diffusion-weighted MRI contrasts, to derive information about blood micro-circulation. In IVIM, information about perfusion of blood is estimated as a pseudo-diffusion process from a PGSE experiment, in which images using low *b*-values are acquired to sensitize the measurement both to diffusion, as well as to perfusion (Le Bihan et al., 1988, 2019; Le Bihan, 1990, 2019). While the contribution to the measured diffusivity from the vascular compartment is an order of magnitude higher than that of diffusion, assuming that they follow the same laws of Brownian motion/ diffusion, they can be estimated from the same microstructure model. Most of the models that have been proposed to quantify such biophysical parameters from diffusion MRI follow a common mixture model formulation as follows:


(1) $$\begin{array}{l} g\left(s,f\right)=\overset{n}{\underset{i=1}{\sum }}f_{i}\varphi \left(s_{i}\right) \end{array}$$

Here, the function *g* aims to give an approximate description of the underlying signal, aiming to find representations for the processes of diffusion and perfusion. This *g* is a convoluted function that models *f* as a linear combination of non-linear functions φ. For the IVIM model, *f* represents the volume fractions associated with perfusion and diffusion and φ holds parameters pertaining to diffusion and perfusion (represented by *s*_*i*_) in Equation (1). Since IVIM works on representing two different processes, we set *n* = 2 for the model which we intend to estimate the parameters of in each voxel of the data. The key underlying idea for using such a model lies in decomposing the signal to a basis (Novikov et al., 2018b) where each of the components of the IVIM model represents a non-exchanging Gaussian compartment. Thus, this gives us a low-rank approximation of the acquired signal with each parameter in the model (*f, s*_1_, and *s*_2_) working as biomarkers for revealing underlying tissue properties and molecular diffusion information. Such a model is difficult to fit, for the following reasons: (a) The exponential components in the signal model are non-orthogonal, which makes it hard to project it along the real-axis by taking an integral and (b) The inherent resolution limit in modeling the exponential decay of multi-exponential models (Istratov and Vyvenko, 1999). The estimation of these model parameters also relies heavily on the range and number of *b*-values used to acquire diffusion MRI data (Lemke et al., 2011; Jalnefjord et al., 2019). It has been shown that if the data are not fit correctly, it can lead to confounding effects such as the pseudo-IVIM effect (Le Bihan, 2019).

While different methods have been proposed to fit this model, the most commonly used method is the segmented fitting approach (Jalnefjord et al., 2018). In this method, initial estimates of the model parameters are fitted by two linear least squares for both the diffusion and perfusion components separately by fixing an *empirical threshold* at a specific *b*-value (Le Bihan et al., 2019). Then using these initial estimates, a form of non-linear regression via Non-linear Least Squares (NLS) solver (typically based on Gauss-Newton and Trust Region based methods) is used. This type of fitting approach is susceptible to different types of issues:

(1) Using estimates from a linear fitting process as done in segmented least-squares fitting, can end up in non-optimal solutions due to factors such as noise and the number of *b*-values pertaining to the diffusion fraction. This issue is also magnified since this type of fitting is sometimes under-determined (depending on the *b*-value distribution from the acquisition). (2) The distributions of *b*-values for IVIM acquisition vary for different organs, which makes it challenging to obtain a heuristic choice for the threshold *b*-value to segregate perfusion from diffusion.

Although prone to the above difficulties in fitting, so far, the segmented fitting has shown to give better results in comparison to Bayesian approaches if handled with care (While, 2017; Jalnefjord et al., 2018). These approaches, in case of estimation degeneracies, discard voxels that violate bounds to and interpolate signal values from the neighboring voxels (Jalnefjord et al., 2018). While different methods have been proposed for fitting IVIM, there has never been a consensus on a standard method which can be used in a generic setting (Le Bihan, 2019). Thus, in this work, we aim to alleviate these issues involved in setting up NLS solvers by setting up a separable inverse problem using variable projection (VarPro). We provide a new topological algorithm for solving this VarPro formulation using simplicial homology Endres et al. (2018) to capture all local minima of the objective function. We do so in the proposed method by constructing a simplicial complex that is homeomorphic to the hypersurface of the objective function, both, in the projected subspace of the VarPro (reduced functional) (Golub and Pereyra, 2003) and the full functional. We discuss the theoretical underpinnings of this algorithm in the section 3. We propose a new method, Topological Projection (TopoPro), to not only make the problem better-conditioned, but also establish a connection between functional analysis and homology groups which makes it well-suited to solve a large class of such problems.

The key issues solved by TopoPro in IVIM estimation are:

1. Spurious solutions obtained due to the presence of multiple global minima in the flat topology of the objective function.
2. Explicit declaration of *b*-value threshold that segregates perfusion from diffusion.
3. Sensitivity toward bounds set for parameters *f*, *D*, and D^*^ for the optimization procedure.

## 2. Approach and Related Work

### 2.1. The Inverse Problem of IVIM

The field of quantifying tissue microstructure is advancing rapidly in terms of developing biophysical models with capabilities to probe a sub-voxel resolution from the measured voxel-averaged signal. These models aim to characterize an ensemble of random walkers at different scales leading to a very challenging inverse problem in terms of parameter estimation. The IVIM model, in this context, aims at doing so by segregating spatial variations caused due to perfusion in the capillary vasculature from the microscopic diffusion of water molecules. Thus, from the measured macroscopic diffusion MR signal, our goal is to estimate successively averaged out dynamics at different spatio-temporal scales and diffusion lengths represented as sums of exponentials for individual signal contributions.

IVIM acquisitions are typically fast scans with *b*-values in the range of 0–1,000 *s*/*mm*^2^, leading to noisy measurements and fewer measurements to estimate the model (Le Bihan et al., 2019). To understand some intrinsic issues with this type of modeling and estimation, we present the overview in **Figure 3**. For each voxel in the brain, we measure noisy signal that may vary according to the *b*-values at which the signal is sampled. Furthermore, it is important to note that the barrier (or threshold) that distinguishes perfusion from diffusion is unknown as it varies according to the organ scanned. Most likely there is no single barrier but a range of values where both diffusion and perfusion are active. Thus, the signal corrupted by noise may push the measurement out of the range of the forward operator φ(*x*), leading to a potential discontinuity in the inverse mapping (φ^−1^(*x*)) from the data space to the model space. Therefore, this violates Hadamard's third condition of well-posed problems (Ivanov, 1988) as the optimizer can lead to drastically different solutions based on the starting point for the optimization process. Like any other inverse problem, the goal of model fitting is to find the inverse mapping φ^−1^(*x*) as it reveals crucial information about the data. In the case of IVIM, the estimated parameters per-voxel, are used as biomarkers for multiple neurological disorders.

### 2.2. Combinatorial Homology Theory

The SHGO algorithm is especially well-suited to overcoming challenges involved in IVIM estimation due to two key theorems that allow for a complete understanding of the parameter hyperspace of the fitting problem. The first theorem deals with extracting locally convex sub-domains from the functions. This allows for the determination of well-defined domains wherein optimal parameters are mathematically guaranteed to be found. The second key theorem deals with invariance, which provides certain guarantees with respect to the total number of optimal parameter sets available in each sub-problem. In order to adequately explain the theoretical foundations of these two theorems a bare minimum nomenclature of several concepts from algebraic and combinatorial topology (Henle, 1994; Hatcher, 2002) is required.

#### 2.2.1. Modulo 2 Homology

**Figure 1:**
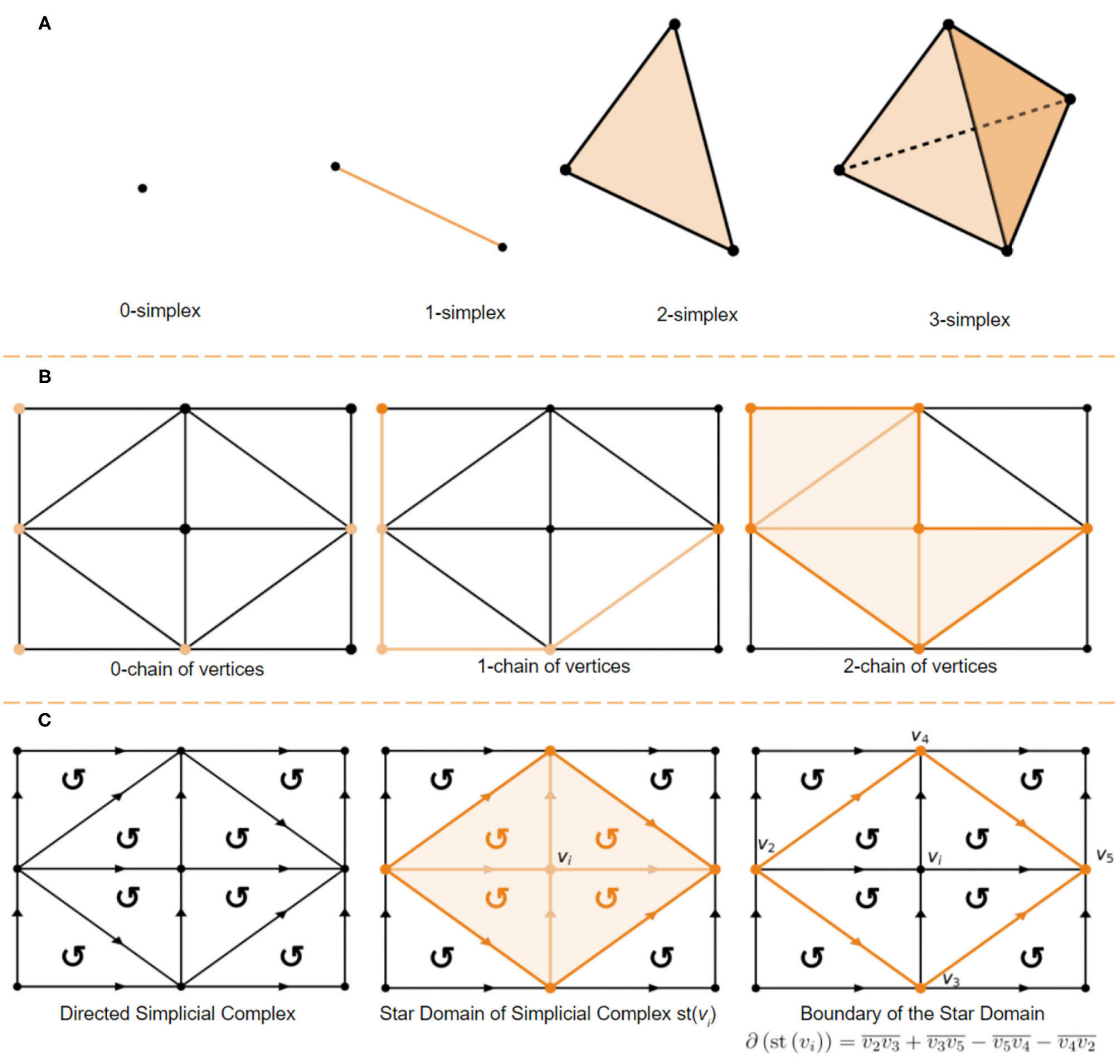
**(A)** 0-simplex (point), 1-simplex (edge), 2-simplex (triangle), and a 3-simplex (tetrahedron). **(B)** 0-chain of vertices, a 1-chain of edges and a 2-chain of simplices. **(C)** Directed 2-simplex in the directed simplicial complex (left), star domain defined by st(*v*_*i*_) (center), and it's boundary defined as $\partial \left(\text{st}\left(v_{i}\right)\right)=\bar{v_{2}v_{3}}+\bar{v_{3}v_{5}}-\bar{v_{5}v_{4}}-\bar{v_{4}v_{2}}$ (right).

A **k-simplex** is a set of *n* + 1 vertices in a convex polyhedron of dimension *n*. A visual demonstration of the first 3-dimensional is provided in Figure 1A. A **simplicial complex** H is a set $H^{0}$ of vertices together with sets $H^{n}$ of *n*-simplices, which are (*n* + 1)-element subsets of $H^{0}$. The only requirement is that each (*k* + 1)-elements subset of the vertices of an *n*-simplex in $H^{n}$ is a *k*-simplex, in $H^{k}$. The subsets of $H^{k}$ are referred to as k-chains, the algebra of k-chains has historical importance in computing the homology group of a surface. A **k**-**chain** is a union of simplices. For example a 0-chain is a set of vertices, a 1-chain is a set of edges and a 2-chain is a set of triangles (Figure 1B provides a visual demonstration) and $C\left(H^{k}\right)$ denotes a *k*−chain of *k*−simplices. Let a vertex in $H^{0}$ be denoted by *v*_*i*_, then, if *v*_*i*_ and *v*_*j*_ are two endpoints of a directed 1-simplex in $H^{1}$ from *v*_*i*_ to *v*_*j*_ then the symbol $\bar{v_{i}v_{j}}$ represents the 1-simplex. This 1-simplex is bounded by the 0−chain $\partial \left(\bar{v_{i}v_{j}}\right)=v_{j}-v_{i}$. A 2-simplex consisting of three vertices *v*_*i*_, *v*_*j*_ and *v*_*k*_ directed as $\bar{v_{i}v_{j}v_{k}}$ has the boundary of directed edges $\partial \left(\bar{v_{i}v_{j}v_{j}}\right)=\bar{v_{i}v_{j}}+\bar{v_{j}v_{k}}+\bar{v_{j}v_{i}}$. The homology theory developed up to this point describes what is known as a **mod 2 homology**. A directed simplicial complex, wherein every volume is signed, as demonstrated in Figure 1B allows us to build an integral homology. For example consider boundary operator acting on a directed simplex shown in Figure 1B the edges of the directed 2-simplex: $\partial \left(\bar{v_{1}v_{2}v_{3}}\right)=\bar{v_{1}v_{3}}-\bar{v_{3}v_{2}}-\bar{v_{2}v_{1}}$. Note that in the **mod 2** homology the 1-chain $\bar{v_{1}v_{3}}+\bar{v_{3}v_{2}}+\bar{v_{2}v_{1}}$ forms a **cycle** and that $\partial \left(\bar{v_{1}v_{3}}+\bar{v_{3}v_{2}}+\bar{v_{2}v_{1}}\right)=\left(v_{3}-v_{1}\right)+\left(v_{2}-v_{3}\right)+\left(v_{1}-v_{2}\right)=\emptyset$. In the directed integral homology we have $\partial \left(\bar{v_{1}v_{3}}-\bar{v_{3}v_{2}}-\bar{v_{2}v_{1}}\right)=\left(v_{3}-v_{1}\right)-\left(v_{2}-v_{3}\right)-\left(v_{1}-v_{2}\right)$ which contains additional information about the path. This is just one example of the trade off between computational complexity and the information retained when using a mod 2 homology vs. a directed integral homology. For example mod 2 homologies fail to distinguish non-orientable surfaces from orientable (ex. klein bottle is non-orientable while a torus is orientable, but they have the same algebraic groups in a mod 2 homology). The **star of a vertex** *v*_*i*_, written st(*v*_*i*_), is the set of points *Q* such that every simplex containing *Q* contains *v*_*i*_ (Henle, 1994; Hatcher, 2002). This concept has a very simple visual demonstration shown in Figure 1C.

#### 2.2.2. Key Theorems for Simplicial Homology Optimization

There are two key theorems developed in Endres et al. (2018), repeated in this section for completion, that are essential for finding all minima in the IVIM problem described in subsection 3.2:

** Theorem 1. Stationary point in a minimizer star domain** *Given a minimizer* $v_{i}\in M\subseteq H^{0}$ *on the surface of a continuous objective function* *f* *with a compact bounded domain in* ℝ^*n*^ *and range ℝ, there exists at least one stationary point of* *f* *within the domain defined by st*(*v*_*i*_).

It is important to note that the domain st(*v*_*i*_) is explicitly computational. The *k*−chain $C\left(H^{k}\right),k=n+1$ of simplices in st(*v*_*i*_) forms a boundary cycle $\partial \left(C\left(H^{n+1}\right)\right)$ with $\partial \left(\partial \left(C\left(H^{n+1}\right)\right)\right)=\emptyset$. The faces of $\partial \left(H^{n+1}\right)$ are the bounds of the domain defined by st(*v*_*i*_) which can be used as explicit in a local optimization algorithm. This explicit computation allows for the fast detection and calculation of the different sets of parameters that are biophysically plausible through sampling of Equation (8). The second key theorem deals with the invariance of problems like Equation (8) (see section 3.2):

** Theorem 2. Invariance of an adequately sampled simplicial complex** H For a given Lipschitz continuous objective function *f* that is adequately sampled by a sampling set of size *N*. If the cardinality of the minimizer pool extracted from the directed simplex H is |M|. Then any further increase of the sampling set *N* will not increase |M|.

In essence, this provides some guarantee that all the plausible solutions of Equation (8) (see section 3.2) are eventually found by SHGO. In addition, it provides the ability to track the number of plausible solutions in higher-dimensional problems where the objective function surface is challenging to visualize.

## 3. Methods

### 3.1. IVIM as a Separable Non-linear Inverse Problem

IVIM, as a biophysical model, aims to capture voxel-averaged bi-modal information through the same PGSE diffusion MRI acquisition. From the signal measurement, we are trying to decompose two processes by approximating them as sums of exponential functions. The IVIM model and its variants (Le Bihan, 2019; Le Bihan et al., 2019), since its introduction, has been represented as a linear combination of two exponential functions. Thus, implicitly we perform a low-rank approximation where each of the diffusion and perfusion compartments of the IVIM model is estimated with separate exponential representation.

This model also relies on the assumption that the perfusion as a process mimics molecular diffusion due to the randomness of the blood vessel network geometry. However, since both work at different scales, we can gain important information from the exponential decay rates of both processes and use them as biomarkers. The standard IVIM model can be expressed in the following bi-exponential form:


(2) $$\begin{array}{l} S/S_{0}=fe^{-bD^{*}}+\left(1-f\right)e^{-bD} \end{array}$$

Here, *e*^−*b*^^D^^*^ is a mono-exponential decay used to represent the perfusion (often mentioned as pseudo-diffusion) process and *e*^−*bD*^ to represent the diffusion component of the signal. Thus, the IVIM can be formulated as a basis set with good approximation properties on compact domains of the measured signal. While different improvements to the model have been suggested (Le Bihan, 2019), we adhere to the standard bi-exponential representation of the model as our goal is to better condition the fitting process and improve estimation accuracy. Therefore, for the IVIM model, we aim to fit three unknowns: *f*, *D* and D^*^. Even if the system of such a model is degenerate, it has often shown to give high bias and poor precision with different methods in the past (Jelescu et al., 2016). We delineate how the proposed method tackles the problem from a topological standpoint and gives a way of resolving these issues with a method that works on smaller separated subspaces. This strategy enables a way of obtaining and evaluating different local minima to improve estimation performance. We can reformulate the non-linear least squares problem mentioned in Equation (1) in the following manner:


(3) $$\begin{array}{l} \underset{x}{\min}\|y-g(s,f){\|}_{2},\;x=\left(\begin{array}{c} s\\ f \end{array}\right)\begin{array}{l} {\}}_{p}\\ {\}}_{q} \end{array},\;p+q=n \end{array}$$

Where *g* represents the non-linear function and *s, f* represent the parameters of the non-linear function that we want to estimate. Here, *f* corresponds to the volume fraction associated with the perfusion component and *s* = *e*^−*bD*^, *e*^−*b*^^D^^*^ can be seen as a set of non-linear parameters. Therefore, for the problem at hand, the solution vector that we intend to find has *n* = 3 (*p* = 2 and *q* = 1). Since we know that ∑*f* = 1, i.e., linear combination of the parameter *f* and want to minimize the *l*_2_ norm, we can rewrite the non-linear function *g*(*s, f*) as


(4) $$\begin{array}{l} r\left(s,f\right)=\|y-\varphi \left(s\right)f{\|}_{2}^{2} \end{array}$$

The columns of the φ(*s*) matrix represent a set of non-linear functions *e*^−*bD*^ and *e*^−*b*^^D^^*^, where φ(*s*) ∈ ℝ^*m* × *p*^ and *m* is the length of the observed variable *y* representing the measured signal. Assuming that we knew *s* in the Equation (4), we could find the linear parameters by simply taking the Moore-Penrose inverse and solve a simple least squares problem as follows:


(5) $$\begin{array}{l} f=\varphi^{†}y \end{array}$$

By doing so, we take the orthogonal projections of the observed measurement matrix *y* onto the range of the variable *f*. Finally, using Equations (1), (4), (5), we can formulate the objective function as:


(6) $$\begin{array}{l} \underset{s}{\min}\|y-g\left(s,f\right)\|=\underset{s}{\min}\|y-\left(\varphi \left(s\right)\varphi {\left(s\right)}^{†}y\right){\|}_{2} \end{array}$$

Thus, we reduce the subspace of the original non-linear least-squares problem by variable elimination.

**Figure 2:**
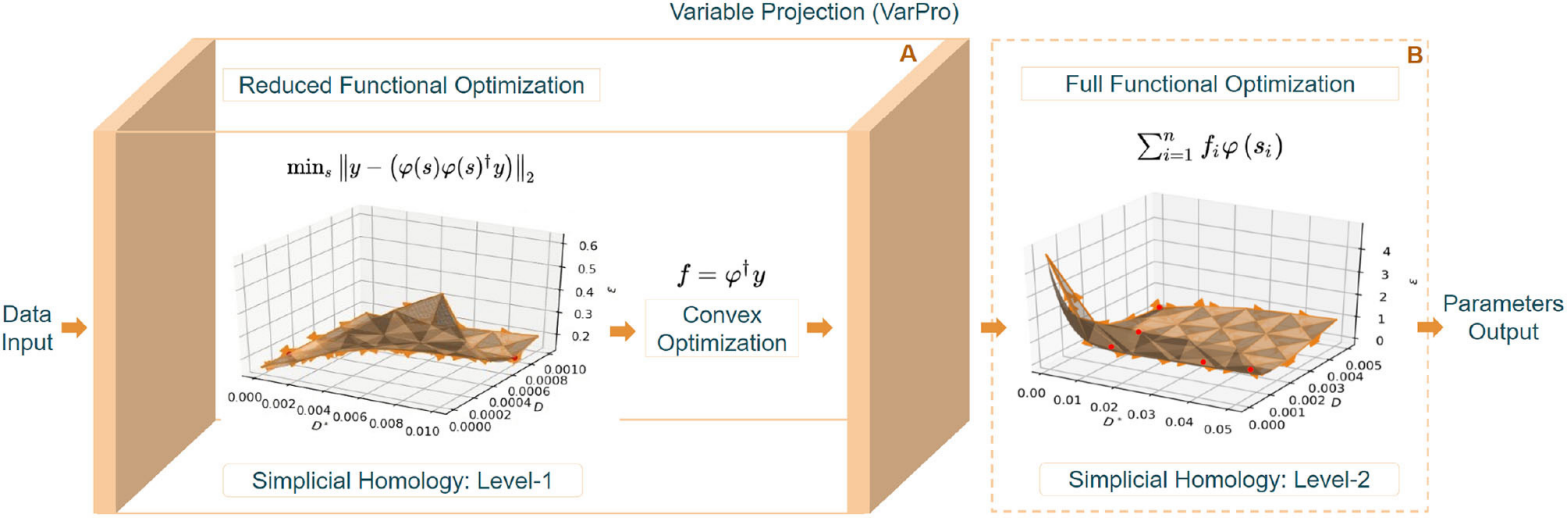
The above figure demonstrates the levels of optimization involved in TopoPro. **(A: Level-1)** Depicts the steps involved in solving the reduced functional and estimating the parameters. The reduced functional of only the non-linear parameters has been visualized with the simplicial homology corresponding to its objective function. **(B: Level-2)** Shows the valley function formed in the process of optimizing the full-functional in the second level. It delineates a problem of bi-modality where the points corresponding to different minima have been indicated in red color.

The proposed algorithm, TopoPro, thus can be seen as a two-level optimization problem shown in Figure 2. We solve for Equation (6) the Variable Projection (VarPro) functional first and then use it to find the values of *f* in Equation (5). Since the VarPro functional is in a reduced subspace, fewer iterations of the non-linear solver (simplicial homology optimizer) used to minimize this are required. Apart from faster convergence, it also yields in better performance due to better conditioning of this joint optimization problem in the projected range of *f* (Golub and Saunders, 1969; Golub and Pereyra, 2003; Pereyra and Scherer, 2019). This causes the minima of the reduced VarPro functional to become better defined than those of the full functional in Equation (1). One key advantage of such a formulation is that it will always converge faster than the full formulation and converges even when the full problem diverges. After estimates of *s* and *f* are obtained in Level-1, they are used to initialize level-2 of TopoPro where we refine the solution to get the final output estimates from the simplicial homology optimizer.

**Figure 3:**
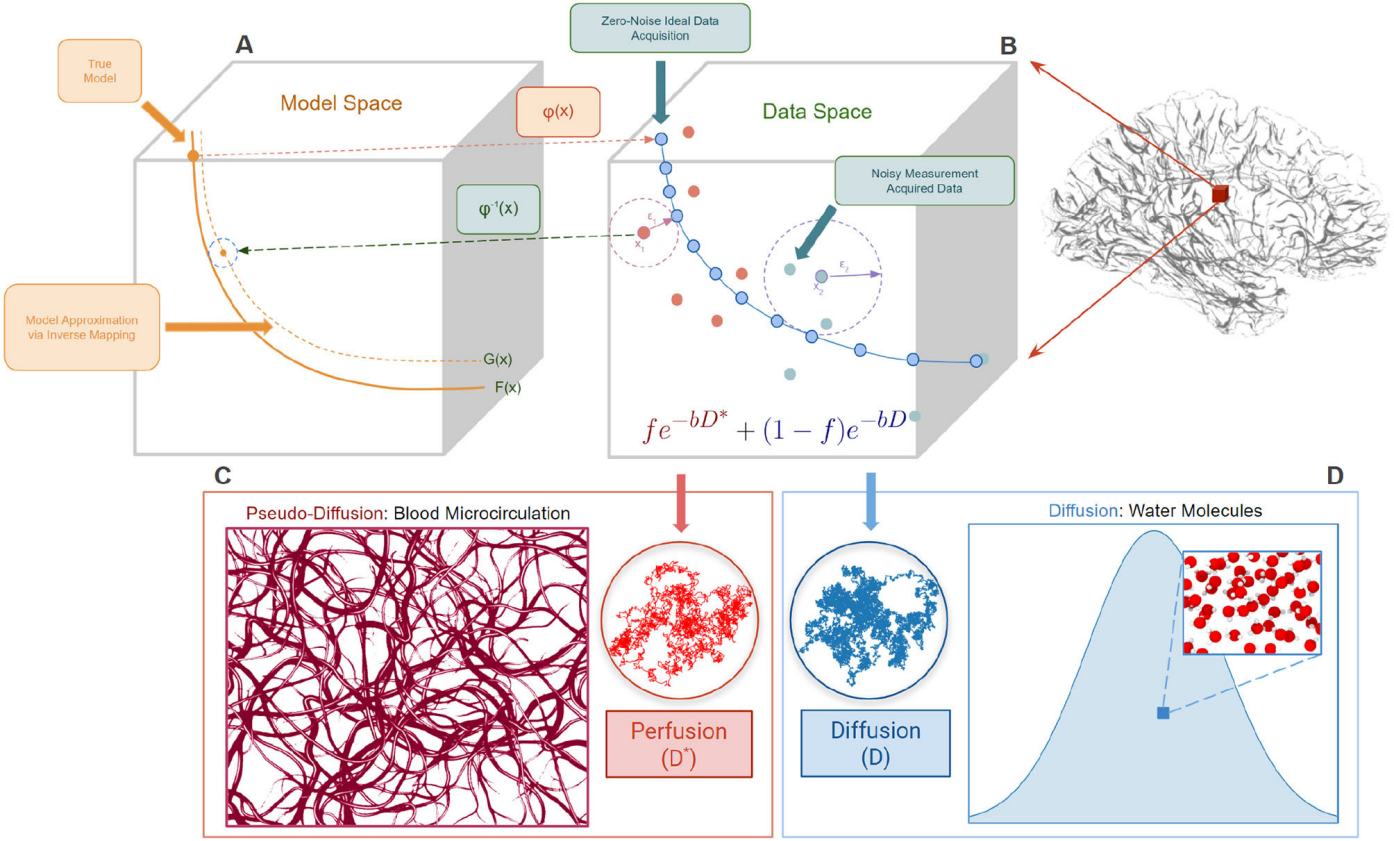
Shows a sketch of the forward mapping φ(*x*) from the true model coordinates in the model space **(A)** to the signal measurements in the data space **(B)** and its inverse denoted by φ^−1^(*x*). **(B)** Notice that some signal measurements in the data space correspond more to perfusion and some correspond more to diffusion. This is a mixing phenomenon. It is important to note that the deviation from the ideal signal is due to different levels of noise (ε). *F*(*x*) represents the true model coordinates in the model-space and *G*(*x*) is the approximation of *F*(*x*) due to noisy measurements. **(C)** Depicts the randomly oriented capillary geometry for the perfusion process which is modeled as a “pseudo-diffusion” process (D^*^). *fe*^−*b*^^D^^*^ is the compartment used to represent this random process. **(D)** Denotes the diffusion process of the water molecules represented by an isotropic compartment *fe*^−*bD*^.

Using VarPro for the bi-exponential model of IVIM improves the conditioning of the problem for the estimating the parameters *f* and D^*^. In the absence of the reduced functional, since the estimation of the linear parameters is dependent on the estimation of non-linear parameters, with iterative gradient-based methods such as NLS, the problem becomes increasingly ill-conditioned (assuming that the parameters converge to the optima). This causes estimation degeneracies as reported in Jelescu et al. (2016).

### 3.2. Simplicial Homology for IVIM Optimization

The simplicial homology global optimization (SHGO) builds on principles from algebraic topology in order to provide insights into both the topology and rigid geometry of objective function hypersurfaces (Endres et al., 2018). In the context of IVIM, these objectives are the non-linear functions φ in Equation (1):


(7) $$\begin{array}{l} \varphi :{\mathbb{R}}^{n}\to \mathbb{R} \end{array}$$

where *n* = 3 for the IVIM model. For global optimization the problem of finding the optimal or “best” parameter fitting can be stated as:


(8) $$\begin{array}{l} \text{minimize}\;\varphi \;\text{, by varying}s_{i}\in {\mathbb{R}}^{n} \end{array}$$

where *s*_*i*_ is the parameter vector components as defined in Equation (1). Due to the non-linear nature of φ, the solution of Equation (8) may contain one or more global minima as well as many local minima. Many difficulties in solving Equation (8) may also arise due to the presence of many minima, saddle points and other complicated features such as infinite valley solutions. The proposed algorithm was developed in order to identify and track all these geometric and topological features while solving a derivative-free optimization problem.

An important distinction between derivative-free optimization algorithms and many gradient-based optimization algorithms is that the former makes different assumptions about the smoothness of the objective function surface such as that defined in Equation (8). On the other hand, derivative-free algorithms assume that no information about the objective function is available, particularly useful in problems where the gradient is unavailable, non-smooth or difficult to compute analytically. Derivative free algorithms rely on deriving information from a sampling of the domain rather using the explicit equations present in the objective function. Finally, while gradient-based optimization algorithms usually only consider the local gradient (and possibly Hessian) information, derivative-free algorithms tend to work with a trade-off between local and global exploration of the surface. During parameter fitting in the IVIM model degeneracy can occur due to bi-modality of the problem (Jelescu et al., 2016; Novikov et al., 2018b). In this case, Equation (8) can no longer be solved using algorithms that rely on local gradient descent. As pointed out in Jelescu et al. (2016) the poor performance of gradient descent methods is due to the presence of multiple local minima that lie within biophysically plausible ranges.

We compare against the other approaches of IVIM fitting on the phantom and the simulated Shepp-Logan Phantom to show better performance of the method. To evaluate the performance, we make use of three different strategies: (a) Normalized Root Mean Squared Error (RMSE) evaluated against the ground truth for different *b*-value distributions in the simulated phantom. (b) Test-retest analysis on data for checking stability and correlation analysis of fitting on the same data. (c) We also compare against other IVIM solvers with quantitative evaluations against the proposed method. The higher performance of the new fitting method allows for a more accurate deduction of the true tissue microstructure using the same diffusion MRI data which, additionally, does not rely on methods such as interpolation (Jalnefjord et al., 2018) which could lead to loss of information in the underlying microstructure.

## 4. Results

We start by discussing the key contribution of the proposed problem in solving the IVIM problem from a parameter estimation standpoint. The main motivation of this work was to stabilize the estimation of the Perfusion Coefficient (D^*^) in the IVIM model as it holds key information about the thermal motion of the blood and perfusion process in the microvascular compartment. This parameter across different literature has been reported to be unstable due to different *b*-value distributions and noise perturbations (Lemke et al., 2011; Fadnavis et al., 2020), leading to reservations against using it. With the help of algebraic topology, we quantitatively uncover the degeneracies involved in the problem with comparisons against available implementations of the most prominent methods: Gauss-Newton type or Levenberg-Marquadt type non-linear least squares solvers, Bayesian estimators via Markov Chain Monte Carlo (MCMC) (Jalnefjord et al., 2018), SHGO (Endres et al., 2018), and MIX (Farooq et al., 2016; Fadnavis et al., 2019). We compare the proposed algorithm on real and simulated data to show drastic improvements in estimation accuracy and stability in model fitting.

### 4.1. Topology of Sums of Exponential Signals With Simplicial Homology

**Figure 4:**
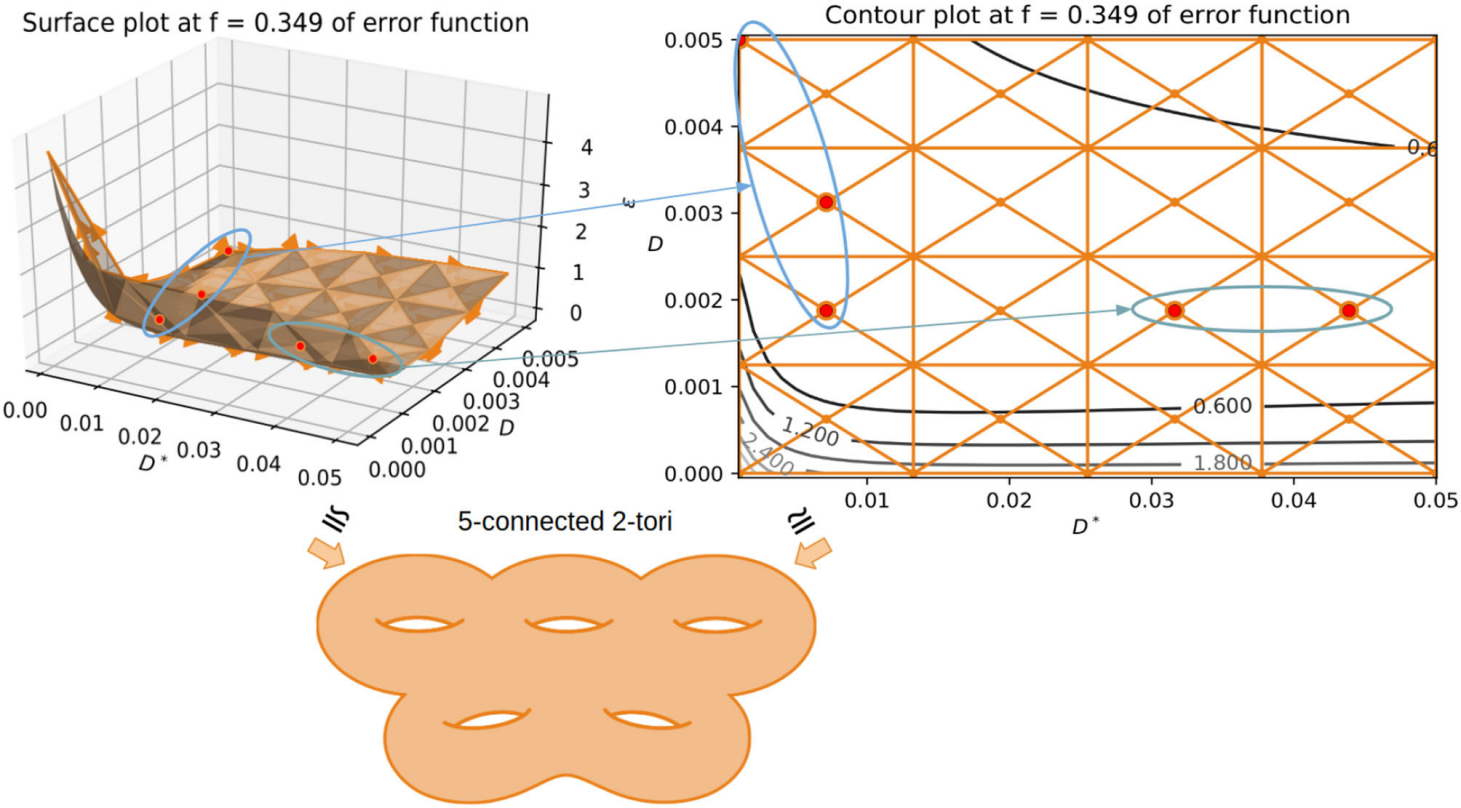
Showing the use of simplicial homology groups to visualize the two biophysically feasible global minima via a surface plot and contour plot. The chain-complex associated with both the minima is highlighted in each case with arrows showing correspondences. Both sub-domains contain a valley of infinite physically plausible solutions for the IVIM problem, one high perfusion and one low perfusion sub-domain. The minima of the depicted objective function surface and its corresponding contour plot is homologous to 5 connected 2-tori.

It has been shown in the past (Jelescu et al., 2016; Novikov et al., 2018b) that the multi-compartment models such as the IVIM model often lead to degeneracies in estimation. This is primarily due to the function being symmetric in nature (having the same exponential representation for each compartment), often leading to two biologically plausible solutions. The objective function surface is very flat leading to a valley of possibly infinite solutions to choose from making the IVIM model an ill-posed (Osullivan, 1986) inverse problem leading to instability and sub-optimal solutions. To alleviate these issues, we propose a framework using simplicial homology-based topological analysis of the solution space (Hatcher, 2002; Endres et al., 2018). Here, we highlight the problem of bi-modality that occurs in fitting the model of IVIM. This has been reported in different literature (Jelescu et al., 2016; Novikov et al., 2018b) in the past, but there has never been a solution to alleviate this problem except when we increase the model complexity with more parameters. In such scenarios, it becomes simpler to extract orientation information from signal transformations and has shown promising results (Novikov et al., 2018c). But in the case of IVIM, such an approach cannot be taken as each compartment of the model is represented with an isotropic basis without encoding any directional information for diffusion or perfusion processes. This causes a problem of identifiability in the solution space when the values of *D* and D^*^ are closer to one another, basically, the voxels where the perfusion is low and the diffusion is high enough to make the parameter scales equivalent. This can be seen by constructing a simplicial complex homologous to the objective function surface of the IVIM model fit to one voxel. As shown in Figure 4, in the process of optimization, when the perfusion fraction *f* has the value of 0.349, two plausible solutions emerge at two very different values. As highlighted in Figure 4 with ellipses around each “valley” sub-domain of local infima (in the surface plot and its corresponding contour plot), one “valley” domain of local infima correspond to low *D* value (~ 0.005 *mm*^2^/*s*) and one to a higher D^*^ (~ 0.04 *mm*^2^/*s*). Hence, this makes it challenging for many optimization algorithms such as segmented, Bayesian, and MIX (Farooq et al., 2016) to settle on a non-spurious minimum. These approaches rely on gradient information to compute the search direction and struggle when the Jacobian has zero entries in a parameter direction. This causes convergence issues such as the algorithm terminating prematurely at an arbitrary D^*^ value in addition to typically being computationally expensive. This choice of D^*^ is what causes fluctuations in the perfusion coefficient maps leading to unstable and high variability in the solutions. The proposed algorithm successively improves the conditioning of the problem using the variable projection functional to estimate the parameters in a reduced subspace using a simplicial homology global optimization step.

### 4.2. Better Conditioning of the Ill-Posed Inverse Problem

Variable Projection (Golub and Pereyra, 2003) has been shown to have successively better conditioning across domains (Fusco et al., 2015, 2016; Farooq et al., 2016; Kurugol et al., 2016). The main advantage is that the reduced functional projects noisy measurements onto the range of the data, making the model estimation process better-posed (Pereyra and Scherer, 2019). Most optimization methods are vulnerable to the starting points of the optimizer, making the inverse mapping harder to estimate. This problem is further exacerbated in the setting of local gradient-based (Gauss-Newton/ Levenberg-Marquardt, Levenberg, 1944) or MCMC style optimization (Gurney-Champion et al., 2018; Jalnefjord et al., 2018).

**Figure 5:**
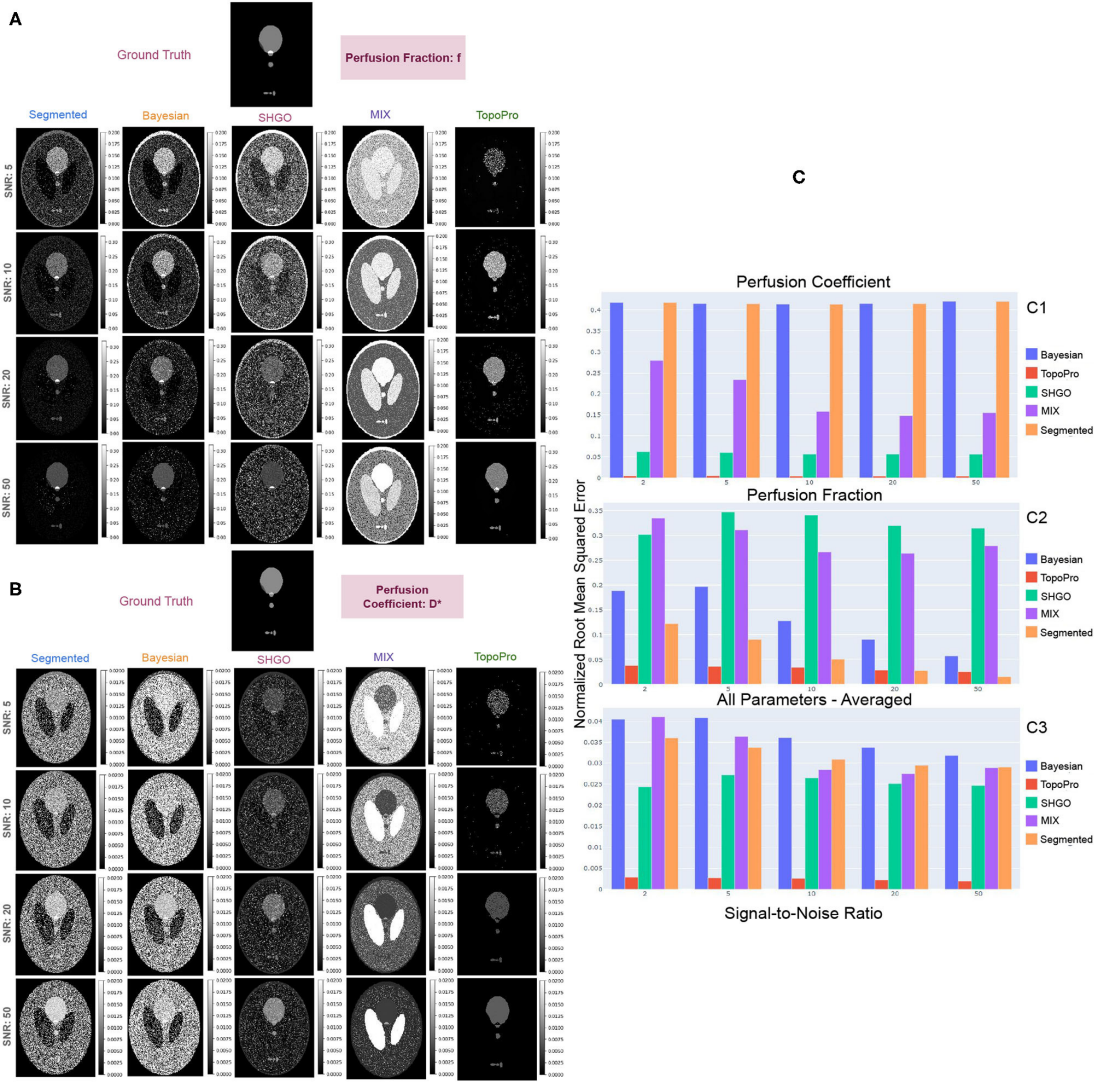
Shows results with the simulated Shepp-Logan phantom for different tumors. In **(A,B)**, we show a qualitative comparison of the simulated data at different SNRs by comparing TopoPro against segmented, Bayesian, SHGO and MIX fitting. The ground truth for each of the model parameters *f* and D^*^ are depicted at the top of their respective estimates with different methods. **(C)** Compares the Root Mean Squared Error of the fits for both D^*^ (C1) and *f* (C2) and its *average* for all model parameters (C3) for each SNR. Notice that TopoPro outperforms the other fitting methods by 10X in the estimation of D^*^.

TopoPro also improves the stability of the algorithm as is evident in Figure 5 even at SNRs as low as 2 and 5. The fluctuations in the *f* and D^*^ parameters is lesser than the standard segmented, Bayesian, SHGO and MIX methods. Thus, not only improves the usability of the parameter maps but also makes the estimation more reliable.

### 4.3. Data Simulation

An IVIM phantom was simulated using the Shepp-Logan digital phantom for six tissues types, here representative *D*, D^*^, and *f* values were assigned to the phantom, including normal tissue, tissue with infiltrating tumor, low-perfused tumor, high-perfused tumor, cavity and cerebrospinal fluid (Fadnavis, 2020b). Diffusion-weighted images (DWI) were simulated using the IVIM model at 54 *b*-values ranging from 0 to 1,000, for five slices, each at a different noise level. To simulate the real-world noise distribution with multi-channel acquisition (i.e., chi-square distribution), a realistic 8-channel coil sensitivity map was used and Gaussian noise was added to the real and imaginary part of each channel of the DWIs respectively. Final DWIs were combined with a sum of square coil combination, and signal-to-noise ratio (SNR) was calculated in the normal tissue on the b0 image. The ground truth was simulated with a 1 × 1 × 1 isotropic resolution (i.e., data of size 256 × 256 × 5 × 54). The same phantom was fitted with segmented Fitting, Bayesian Fitting, SHGO, MIX and the proposed TopoPro algorithms. The phantom was simulated such that each slice has a different SNR: 2, 5, 10, 20, and 50 particularly. The goal here was to examine the stability of the optimization routines in the different algorithms from a qualitative and quantitative standpoint.

### 4.4. Comparisons With Simulated Data

While we can see that the parameter maps obtained from TopoPro are a stable and visually more informative as compared to the other methods, they are also much closer to the ground truth as shown in Figure 5. We evaluated the estimates of each of the parameters using the normalized Root Mean Squared Error (RMSE) (Pedregosa et al., 2011). From the bar chart of RMSE scores in Figure 5, we can infer that the performance in the estimation of diffusion is similar to one another, thus causing the lines to overlap onto one another. For the perfusion coefficient parameter (D^*^), we see a trend of a slight increase with an increase in the Signal-to-Noise Ratio (SNR). The Bayesian and segmented fitting approaches show similar performance and do well in the estimation of the parameter *f*, but fluctuate and give unstable estimates for the parameter D^*^. SHGO and MIX, on the other hand, perform consistently better as compared to segmented and Bayesian fitting for D^*^. However, it can be seen that TopoPro has a 10 × lower RMSE as compared to the other methods when estimating the D^*^ and *f* parameters, with a steady decrease with an increase in the SNR. It is also important to note that the estimates of the parameters are a lot more stable at SNRs 5 and 10, with no fluctuations in the areas where there is no tissue micro-environment. For instance, if we look at the D^*^ map, it can be seen that even where there is no data, most optimization strategies would find values higher than 0.02 and *f* > 0.2. This effect could further be amplified in real data where the ground truth is unknown.

### 4.5. Comparisons With Real Data

**Figure 6:**
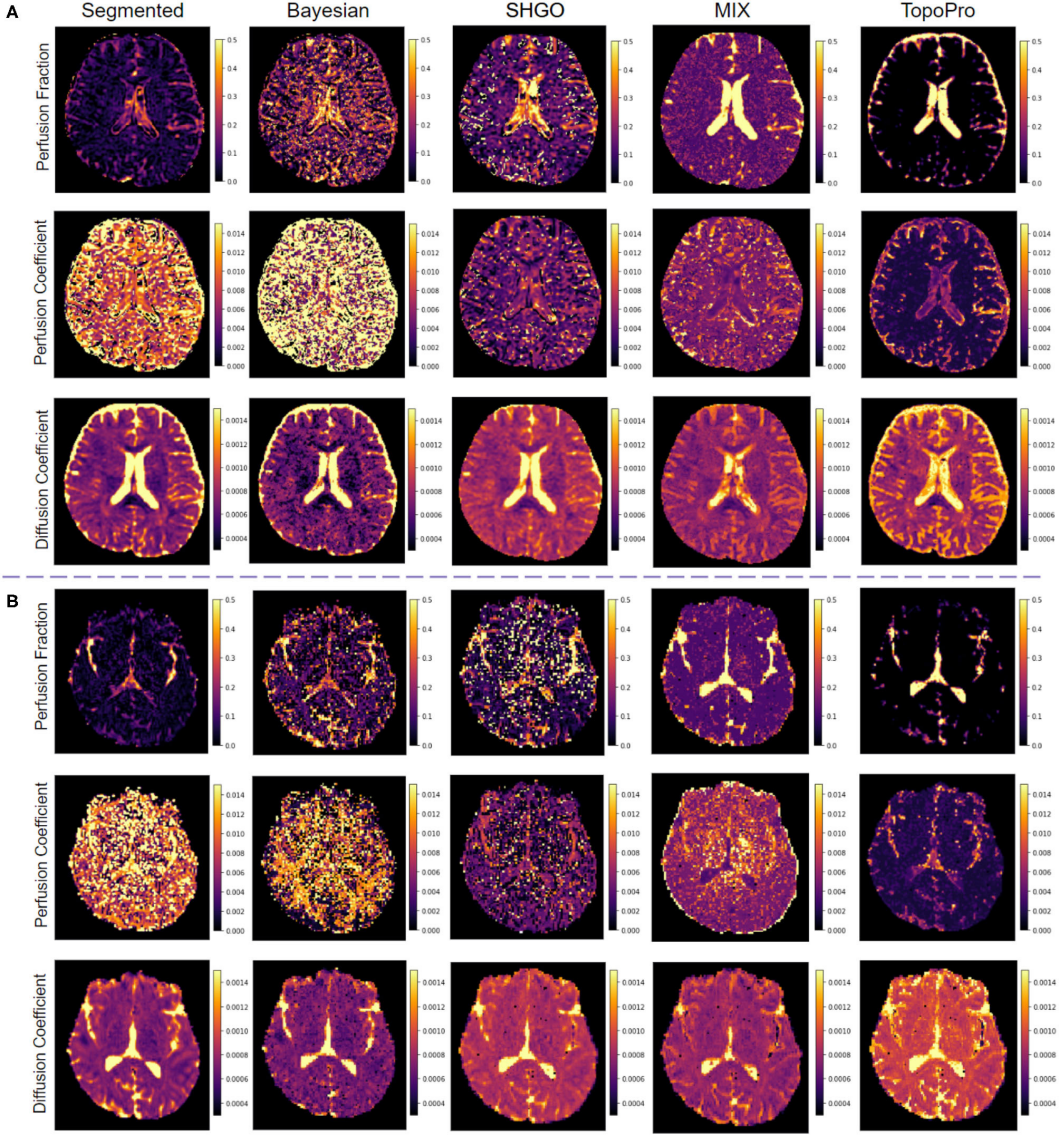
Comparisons of TopoPro on two different real human brain datasets vs. 4 different methods: **(A)** DWI dataset with dimensions: 256 × 256 × 54 × 21 - slice 33 and **(B)** DWI dataset with dimensions: 140 × 140 × 84 × 49 - slice 42. Notice how TopoPro provides improved estimates of the different parameters of the IVIM model in both data. The results are compared against methods: Segmented, Bayesian, SHGO, and MIX.

In Figure 6, we show the results on two real subjects with different *b*-value distributions. Typically, IVIM is a fast acquisition and has *b*-values ranging from 0 to 1,000. In Figure 6, we compare the fitting of TopoPro against Bayesian, segmented, SHGO and MIX Fitting. The data has 21 *b*-values with a voxel resolution 0.938 × 0.938 × 2.5 in Figure 6A (Peterson, 2016) and has 49 *b*-values with voxel resolution 1.714 × 1.714 × 1.7 in Figure 6B (Fadnavis, 2020a). As it can be seen from both Figures 6A,B, TopoPro performs better than the other four methods from a qualitative standpoint with lesser visual fluctuations in the estimated values of D^*^ and *f*. The D^*^ values for Bayesian and the segmented fitting are wrongly estimated for a large number of the voxels. It is important to note that the TopoPro algorithm always finds a better approximation of the parameter values as compared to the other approaches. It is also important to note that by construction, the segmented and Bayesian fitting seems to work in the estimation *f* and *D* but fail to fit for D^*^. The reason for this is that MCMC and local gradient-based Gauss-Newton/Levenberg-Marquardt algorithms cannot find a global minimum in the infinite solution space of the flat valley like surface of the objective function. This causes the algorithms to often find a spurious minima as the estimate leading to noisier maps. It is also important to note that both Bayesian and segmented fitting depend heavily on the starting points of the optimizer and the bounds on the parameters. To initialize the segmented fitting, the standard linear least squares approximation was used as the starting points and for the Bayesian fitting, the initial values used were obtained from the segmented Fitting. For the Bayesian estimation, we set the burn-in to 1,000 and used the flat prior. Another key aspect of the both Bayesian and segmented algorithms is that the voxels which do not obey the bounds need to be discarded and values for these voxels are typically interpolated with a nearest neighbor algorithm. For both the Bayesian and segmented fitting, the bounds used were: *f*∈[0, 0.9], D^*^∈[0, 0.1] and *D*∈[0, 0.004]. The *b*-value threshold used for segregating the perfusion and diffusion component was 200.

### 4.6. Topological Analysis for Stability: Test-Retest

**Figure 7:**
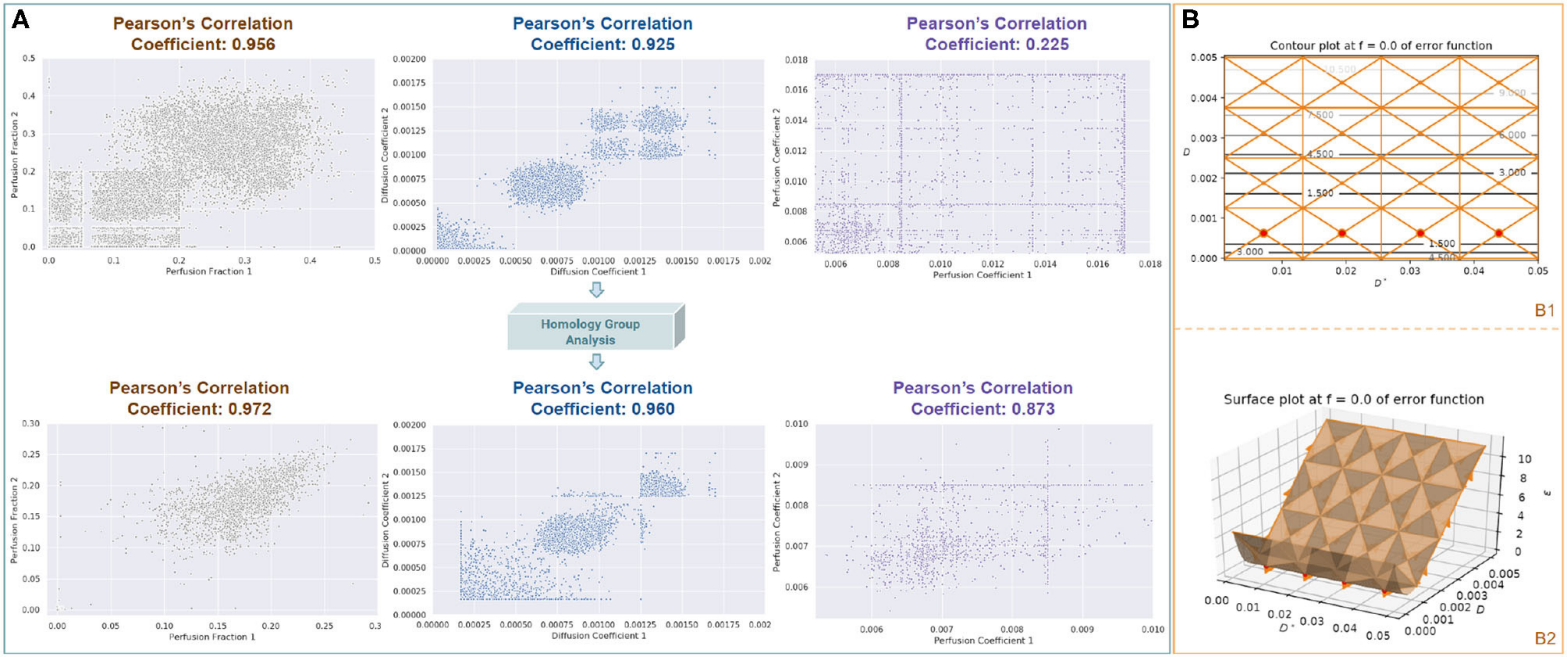
**(A)** Here, we demonstrate the test-retest reliability of the TopoPro algorithm. On the same data, we split the data in half by choosing alternating *b*-values. We show using an inverse penalty function that we can improve the stability across all parameters. We plot the first half of the data-split along Y-axis and the other along X-axis. Notice how the reliability (Pearson Correlation Coefficient) improves across all parameters calculated for each parameter, i.e., Perfusion Fraction (*f*), Diffusion Coefficient (*D*), and Perfusion Coefficient (D^*^). **(B)** We use the simplicial homology optimizer to show the infinite solution space of D^*^ at a particular value of *D* when *f* = 0. This has been depicted with the surface plot of simplicial complex **(B1)** and its corresponding contour plot **(B2)**. Notice that all values in the solution subspace for D^*^ are equally good in this case, making it hard for the optimizer to find a solution.

Topological analysis allows us to gain insight into the objective function surface and enables us to provide the algorithm with this information to find more physically meaningful solutions and speed up the optimization process. For example, consider the objective function surface at a low perfusion voxel when the perfusion fraction is held at a constant zero then there is an infinite number of optimal solutions for D^*^ as shown in Figure 7B1,2. This justifies adding additional constraints in the physically feasible sub-domain, for example by adding the non-linear penalty function *g*(*x*) = (^D^^*^)2 ⇔*f* ≤ 0.2 which not only speeds up gradient descent sub-routines in the valley sub-domains but also allows the algorithm to search for the most meaningful global minimum to use in the final result. From the information automatically extracted by the simplicial homology optimization routine, we can add yet more sophisticated modifications to the objective function that apply in different sub-domains of the objective function depending on both the learned mathematical and the physical interpretation of the problem at a particular voxel. In the first step of VarPro, we obtain two sub-domains which contain an infinite number of physically feasible solutions. By analyzing both the domains simultaneously using simplicial homology optimization, we can choose the best sub-domain for a given voxel and adapt penalty functions to find optimal parameter vectors in the respective star domains.

In order to show the stability of the proposed method, we perform test-retest reliability on the data as shown in Figure 7A. To do so, we split the data into two halves along the *b*-values of the DWI image. This is done by down-sampling the *b*-values such that we choose alternating *b*-values to split the data. Then we fit the IVIM model to each half and calculate the Pearson's correlation coefficient for each parameter. We show that the performance of the TopoPro optimizer can be improved by using a penalty function by using the constraints derived from the topological analysis. It is important to note that the stability of the D^*^ parameter improves drastically as seen in Figure 7A (correlation coefficient increased by 0.65) on restarting the simplicial homology optimizer and using the inverse penalty function. The increased reliability is due to selecting the appropriate penalty functions in each detected physically feasible sub-domain using simplicial homology optimization. Once the most appropriate sub-domain for a particular voxel sub-problem has been found, the appropriate penalty constraints can be applied in the objective function to avoid difficulties with ill-defined infinum sub-domains.

## 5. Discussion

In this paper, for the first time, we propose a topological solver for optimizing separable non-linear inverse problems, such as fitting sums of exponential functions to the data. We showed how it is useful for solving the ill-posed problem of estimating IVIM parameters commonly represented as a bi-exponential model. We delineate how simplicial homology can be used as a computational tool to study the topology of the objective function surface and the difficulties involved in this process. The proposed method can be used as a black-box solver and alleviates issues of declaring explicit bounds for different data sets and *b*-value distributions. We compare against other open-source implementations of other standard approaches to fit the data and show how TopoPro outperforms them by 10 × improvement in estimation accuracy. The proposed framework mitigates the crucial problems of noise tolerance and bi-modality via a combination of variable projection and simplicial homology global optimization, leading to improved conditioning of the problem and better initialization. Lastly, we compare the model fitting methods on both simulated and real data showing significant improvements in terms of accuracy (RMSE) and stability (test-retest) with the TopoPro method. The method performs well even though each compartment of the IVIM is isotropic and no orientation information is available to improve identifiability as in the case of more complex microstructure models. Our method provides a unique capability of dealing with multiple global minima in the solution space and avoiding fluctuations in the parameter maps.

## Data Availability Statement

The original contributions presented in the study are included in the article/Supplementary Material, further inquiries can be directed to the corresponding author/s.

## Author Contributions

SF, SE, and EG conceived the research and conducted the analysis. QW, Y-CW, HC, SK, SR, and AR helped with data and design of the analyses. All authors wrote the manuscript.

## Funding

SF, EG, AR and SK were supported by the National Institute of Biomedical Imaging And Bioengineering (NIBIB) of the National Institutes of Health (NIH) under Award Number R01EB027585. SR was supported by University of Washington's Royalty Research Fund. QW and YW were supported by NIH grants R01 AG053993 and R01 NS112303. SE acknowledges the support from the German Research Foundation (DFG) research training group MIMENIMA (GRK 1860). AR was also funded through a grant from the Alfred P. Sloan Foundation and the Gordon & Betty Moore Foundation to the University of Washington eScience Institute Data Science Environment.

## Conflict of Interest

The authors declare that the research was conducted in the absence of any commercial or financial relationships that could be construed as a potential conflict of interest.

## Publisher's Note

All claims expressed in this article are solely those of the authors and do not necessarily represent those of their affiliated organizations, or those of the publisher, the editors and the reviewers. Any product that may be evaluated in this article, or claim that may be made by its manufacturer, is not guaranteed or endorsed by the publisher.
