# Supplementary material for: Bifurcated Topological Optimization for IVIM
Source: Front Neurosci. 2021 Dec 15;15:779025. doi: 10.3389/fnins.2021.779025 (PMC8714828; doi:10.3389/fnins.2021.779025)
Supplement: Supplementary file 1 [file Data_Sheet_1.pdf]

Supplementary Material

1 COMBINATORIAL FIXED POINT THEORY

Sperner's lemma presented in the seminal work [Sperner (1928)] can be considered as the combinatorial analog of fixed point theory. The discrete Sperner's lemma [Sperner (1928)] can be connected to traditional fixed point theories such as Brouwer's fixed point theorem [Brouwer (1911)] as shown for example in the proof contained in [Henle (1979)]. Recently there have been several developments in the extension of this lemma generalising the theorem to more sapces. A major advancement was accomplished by De Loera, Peterson and Su [De Loera et al. (2002)] where they proved the Atanassov conjecture [Atanassov (1996)] which states that for any polytope with N vertices there are $N - n$ simplices that receive a complete set of Sperner labels. Meunier et. al. [Meunier (2006)] further extended this theorem and more recently Musin [Musin (2015)] extended the theorems to a large class of manifolds with or without boundary. The theorems by Meunier and Musin allow us to extend Sperner's lemma to a simplicial complex built in a $(n+1)$-dimensional non-euclidean space. The relation between the smooth and discrete fixed point theories is demonstrated visually in Fig. S1.

2 THEORY OF SIMPLICIAL HOMOLOGY GLOBAL OPTIMIZATION

The recently developed simplicial homology global optimization (SHGO) method allows us to apply powerful theorems from the field of algebraic topology to the numerical field of global optimization [Endres et al. (2018)] in order to provide insights into understanding both the topology and rigid geometry of objective function hypersurfaces. In this context we define a general optimization problem of the form:

$$\min_{\mathbf{x}} f(\mathbf{x}) \\ \text{s.t. } \mathbf{g}(\mathbf{x}) \geq 0 \tag{S1}$$

the Lipschitz continuous real objective function $f : \mathbb{R}^n \rightarrow \mathbb{R}$ maps a vector of dimension n to a scalar value. The parameter set $\mathbf{x}$ (also called decision variables in this context) are assumed to be bounded. The vector-valued function g maps the set of constraints $\mathbf{g} : [\mathbf{l}, \mathbf{u}]^n \rightarrow \mathbb{R}^m$, for example if lower and upper bounds l_i and u_i are implemented for each variable then we have an initially defined hyperrectangle

$$\mathbf{x} \in \Omega \subseteq [\mathbf{l}, \mathbf{u}]^n = [l_1, u_1] \times [l_2, u_2] \times \ \dots \ \times [l_n, u_n] \subseteq \mathbb{R}^n \tag{S2}$$

where Ω is the limited feasible subset excluding points outside the bounds and constraints.

$$\Omega = \{\mathbf{x} \in [\mathbf{l}, \mathbf{u}]^n \mid \mathbf{g}_i(\mathbf{x}) \geq 0, \forall i = 1, \dots, m\} \tag{S3}$$

A key construction involves a simplicial complex approximation $\mathcal{H}$ of the objective function hypersurface $f : \mathbb{R}^n \rightarrow \mathbb{R}$ found using a triangulation $\mathcal{K}$ of (arbitrary, but intelligently chosen) sampling points $\mathcal{P}$ on the objective function hypersurface. This construction maps a special directed simplicial complex defined by a composition mapping $h \circ f : \mathcal{P} \rightarrow \mathcal{H}$. We can then use the construction $\mathcal{H}$ in two key applications involving (i) the Invariance of the objective function surface which describes a homological group (and in particular the rank of the homology groups $\mathbf{H}_i(\mathcal{H})$) and (ii) the computation of locally convex sub-domains containing one or more minima of the objective function which allows for the generation of sub-problems

that can be solved using local optimization methods. These concepts are used in order to understand both the topology and the geometry of the problem described in S1 which in turn leads to efficient algorithms for computing the global minima f^* and the optimal parameter set $\mathbf{x}^*$. An overview of how these concepts are related can be demonstrated in the following diagram:

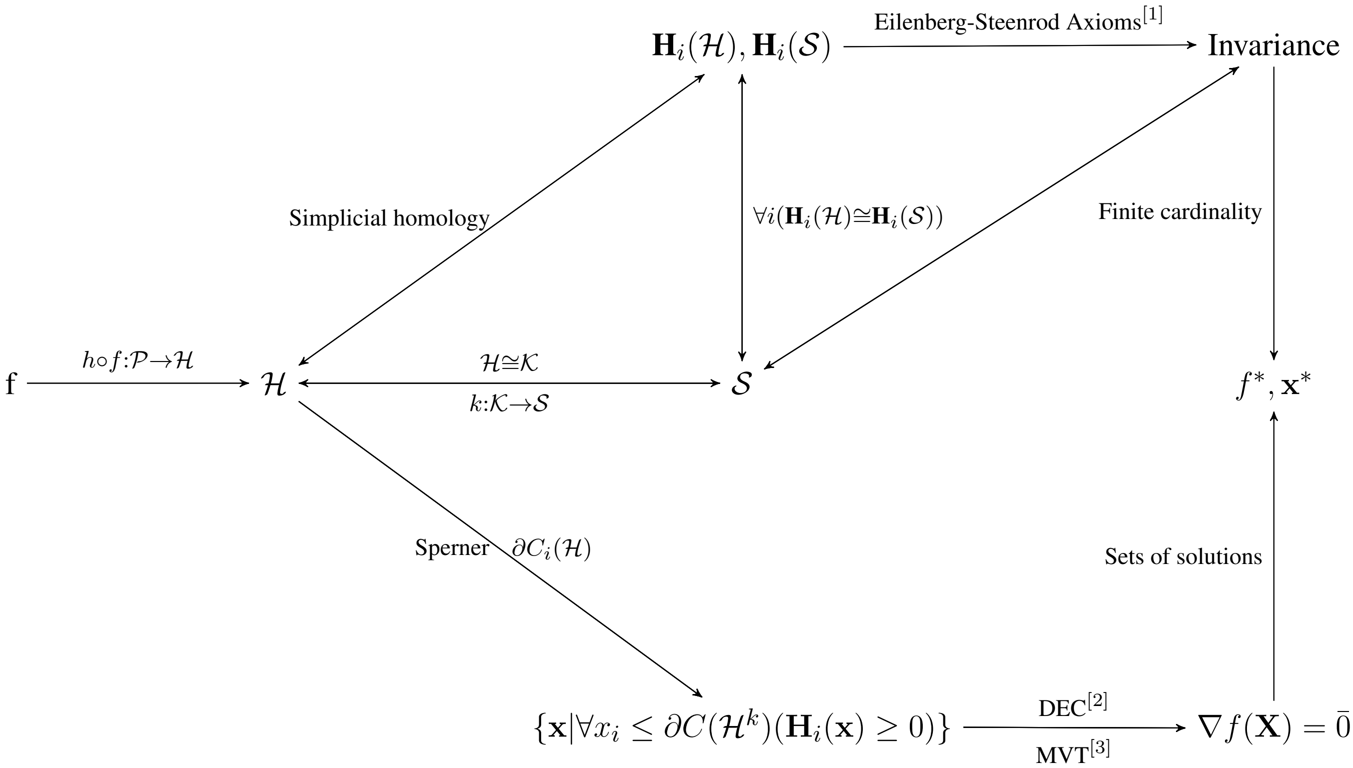

1. Eilenberg-Steenrod Axioms Eilenberg and Steenrod (1952)
2. Discrete Exterior Calculus
3. (Discrete) Mean Value Theorem

The mapping $h \circ f$ The mapping h defines the constructions used to build the simplicial complex $\mathcal{H}$ on the hypersurface f from which we compute the cardinality of the homology groups. The set of all 0-chains $\mathcal{H}^0 := \mathcal{P}$ is the set of all vertices of $\mathcal{H}$ built from the set of feasible sampling points $\mathcal{P} = \{\mathbf{x} \in \mathcal{X} \mid \mathrm{g}(\mathbf{x}) \geq 0\}$. The simplicial complex $\mathcal{H}$ is constructed by a triangulation connecting every vertex in $\mathcal{H}^0$. The set $\mathcal{H}^1$ is constructed by directing every edge. The edge is directed as $\overline{v_i v_j}$ from v_i to v_j iff $f(v_i) < f(v_j)$ so that $\partial\left(\overline{v_i v_j}\right) = v_j - v_i$. Similarly an edge is directed as $\overline{v_j v_i}$ from v_j to v_i iff $f(v_i) > f(v_j)$ so that $\partial\left(\overline{v_j v_i}\right) = v_i - v_j$. We let the higher dimensional simplices of $\mathcal{H}^k, k = 2, 3, \ldots n+1$ be directed in any arbitrary direction (it is only important they are assigned some direction) which completes the construction of the complex $h \circ f : \mathcal{P} \to \mathcal{H}$. We can now use $\mathcal{H}$ to find the minimiser pool for the local minimisation starting points used by the algorithm:

DEFINITION 1. *A vertex v_i is a minimiser iff every edge connected to v_i is directed away from v_i, that is $\partial\left(\overline{v_i v_j}\right) = (v_{j \neq i} - v_i) \vee 0 \; \forall v_{j \neq i} \in \mathcal{H}^0$. The minimiser pool $\mathcal{M}$ is the set of all minimisers.*

This definition of a minimiser in this context is a strictly discrete approximation and should not be confused with the exact solutions $\mathbf{x}^*$ that are sometimes referred to as minimisers in literature. The cardinality of the set $\mathcal{M}$ has an important equality with the rank of the homology groups $\mathbf{H}_i(\mathcal{H})$ as

described in [Endres et al. (2018)] and can be computed efficiently without the need to explicitly compute all the homology groups of the surface.

Since the equality described in the previous paragraphs allows for a fast computation of the rank of the homology groups $\mathbf{H}_i(\mathcal{H})$ we can use to invoke Invariance theorems from algebraic topology. A persistent challenge in optimization is the presence of so-called ”valleys” in non-convex functions epitomised by the popular Rosenbrock function Rosenbrock (1960). A related issue is the presence of a sub-domain of containing an infinum of ”equally good” solutions. In our approach we address this issue by relaxing the assumption that S1 contains a minimum, and instead contains one or more infima (including sub-domains that possibly contain an infinite number of solutions), this statement is written as follows:

$$\inf_{\mathbf{x}} f(\mathbf{x}) \\ \text{s.t. } \mathbf{g}(\mathbf{x}) \geq 0 \tag{S4}$$

LEMMA 1. *The Invariance extends to hypersurfaces containing non-finite sub-domains containing an infinum of f by replacing 1 with the following definition:*

DEFINITION 2. *A 1-chain of $C(\mathcal{H}^1)$ is a minimiser iff it contains a 1-chain $C(\mathcal{H}^1)$ where for every alternating vertex v_i in the 1-chain, every edge connected to v_i is directed away from v_i, that is $\partial\left(\overline{v_i v_j}\right) = (v_{j\neq i} - v_i) \vee 0 \; \forall v_{j\neq i} \in \mathcal{H}^0$. The minimiser pool $\mathcal{M}$ is the set of all minimisers.*

Proof: Note that the algebraic groups $\mathbf{H}_k(\mathcal{H}) \cong \mathbf{H}_k(\mathcal{K}) \cong \mathbf{H}_k(\mathcal{S}) \; \forall k \subset \mathbb{Z}$ are closed groups by definition, therefore only the addition of new elements in the group affect the rank of the groups. The groups $\mathbf{H}_0(\mathcal{H}) \cong \mathbf{H}_0(\mathcal{K}) \cong \mathbf{H}_0(\mathcal{S})$ are known to contain just two elements (all the cycles with an even number of vertices and all the cycles with an odd number of vertices) and are therefore homologous to the groups *Theorem 1 (refer main document)*. Let $C(\mathcal{H}^k)$ be a k-chain $\mathcal{H}_k$ and let $C_1, C_2 \in C(\mathcal{H}^1)$ be any two 1-chains. Since $\partial\left(C_1 + C_2\right) = \partial\left(C_1\right) + \partial\left(C_2\right)$ we do not add additional elements that can increase the rank of the groups $\mathbf{H}_1(\mathcal{H}) \cong \mathbf{H}_1(\mathcal{K}) \cong \mathbf{H}_1(\mathcal{S})$ by refining either of the chains C_1, C_2. Similarly groups with $k > 1$ remain unchanged and therefore have an equivalent invariance. Thus we have simply extended the concept of a finite point, or vertex, containing a minimum to a larger sub-domain of higher dimensional objects. This sub-domain is, in general, still contained in Euclidean $\mathbb{R}^n$ space since the star of the chain contains one or more n-simplices.

3 DIFFUSION COEFFICIENT IN IVIM

One of the motives of IVIM is to improve the estimation of the diffusion coefficient (D) as the diffusion acquisition is susceptible to both diffusion and blood microcirculation. In this scenario, IVIM can be perceived as a correction mechanism to improve the fitting of D by disentangling the perfusion (pseudo-diffusion) effect at lower b-values in the acquisition. It is important to note that, the primary purpose of IVIM is to focus on the estimation f and D^* parameter. However, the estimation of D is generally stable across all methods as depicted in Fig. S2. As it can be seen in the simulated and real data in the results section of the main text, overfitting for the parameter D leads to fluctuations in the parameters D^* and f. TopoPro on the other hand, finds an optimal trade-off in the estimation of all parameters: D, D^* and f.

4 FIGURES

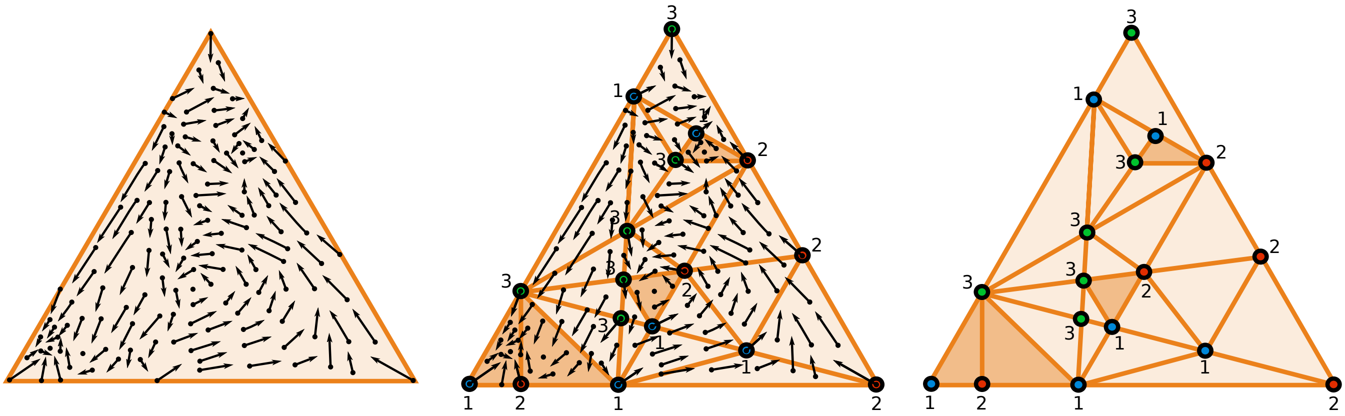

Figure S1: A smooth vector field, such as a gradient field, can be approximated by a triangulation of sampling points in this field. After labeling each vertex to be associated with an $n + 1$ direction in the vector field it becomes possible to search for a Sperner labeling. This special type of labeling represents a combinatorial analogue of a fixed point (local optimum). Within each Sperner labeling a fixed point in the approximated smooth field is guaranteed to be found through Brouwer's Theorem.

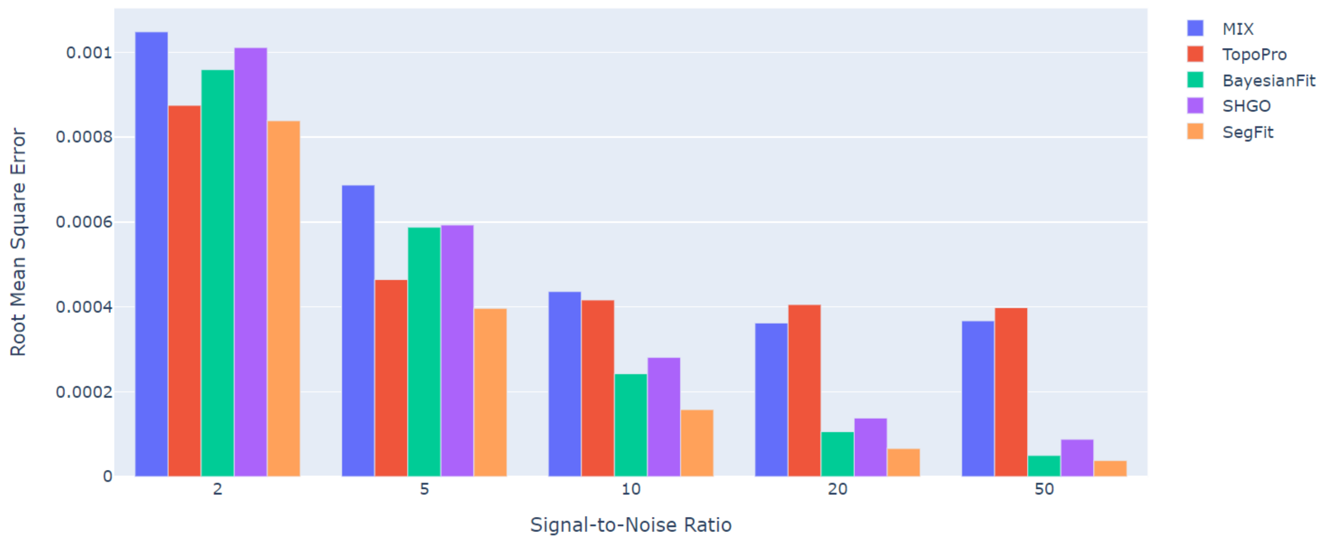

Figure S2: The above bar chart depicts the normalized root mean squared errors from fitting the phantom at Signal-to-Noise Ratios 2, 5, 10, 20 and 50 in the estimation of Diffusion Coefficient: D. Note that the errors across different methods have similar order of 10^{-2}, showing stability for all methods in the estimation of D. However as we saw in the main document stability for D^* and f is only guaranteed using TopoPro.

REFERENCES

Atanassov, K. (1996). On sperner's lemma. *Studia Scientiarum Mathematicarum Hungarica* 32, 71–74

Brouwer, L. E. J. (1911). Über Abbildung von Mannigfaltigkeiten. *Mathematische Annalen* 71, 97–115

De Loera, J. A., Peterson, E., and Edward Su, F. (2002). A Polytopal Generalization of Sperner's Lemma. *Journal of Combinatorial Theory, Series A* 100, 1–26

Eilenberg, S. and Steenrod, N. (1952). Foundations of algebraic topology. *Mathematical Reviews (MathSciNet): MR14: 398b Zentralblatt MATH, Princeton* 47

Endres, S. C., Sandrock, C., and Focke, W. W. (2018). A simplicial homology algorithm for lipschitz optimisation. *Journal of Global Optimization* 72, 181–217. doi:10.1007/s10898-018-0645-y

Henle, M. (1979). *A Combinatorial Introduction to Topology* (Unabriged Dover (1994) republication of the edition published by WH Greeman & Company, San Francisco, 1979)

Meunier, F. (2006). Sperner labellings: A combinatorial approach. *Journal of Combinatorial Theory, Series A* 113, 1462 – 1475. doi:http://dx.doi.org/10.1016/j.jcta.2006.01.006

Musin, O. R. (2015). Extensions of Sperner and Tucker's lemma. *Journal of Combinatorial Theory, Series A* 132, 172–187

Rosenbrock, H. H. (1960). An Automatic Method for Finding the Greatest or Least Value of a Function. *The Computer Journal* 3, 175–184. doi:10.1093/comjnl/3.3.175

Sperner, E. (1928). Neuer beweis für die invarianz der dimensionszahl und des gebietes. *Abhandlungen aus dem Mathematischen Seminar der Universität Hamburg* 6, 265
