# Supplementary figures and images for: Bifurcated Topological Optimization for IVIM
Source: Front Neurosci. 2021 Dec 15;15:779025. doi: 10.3389/fnins.2021.779025 (PMC8714828; doi:10.3389/fnins.2021.779025)

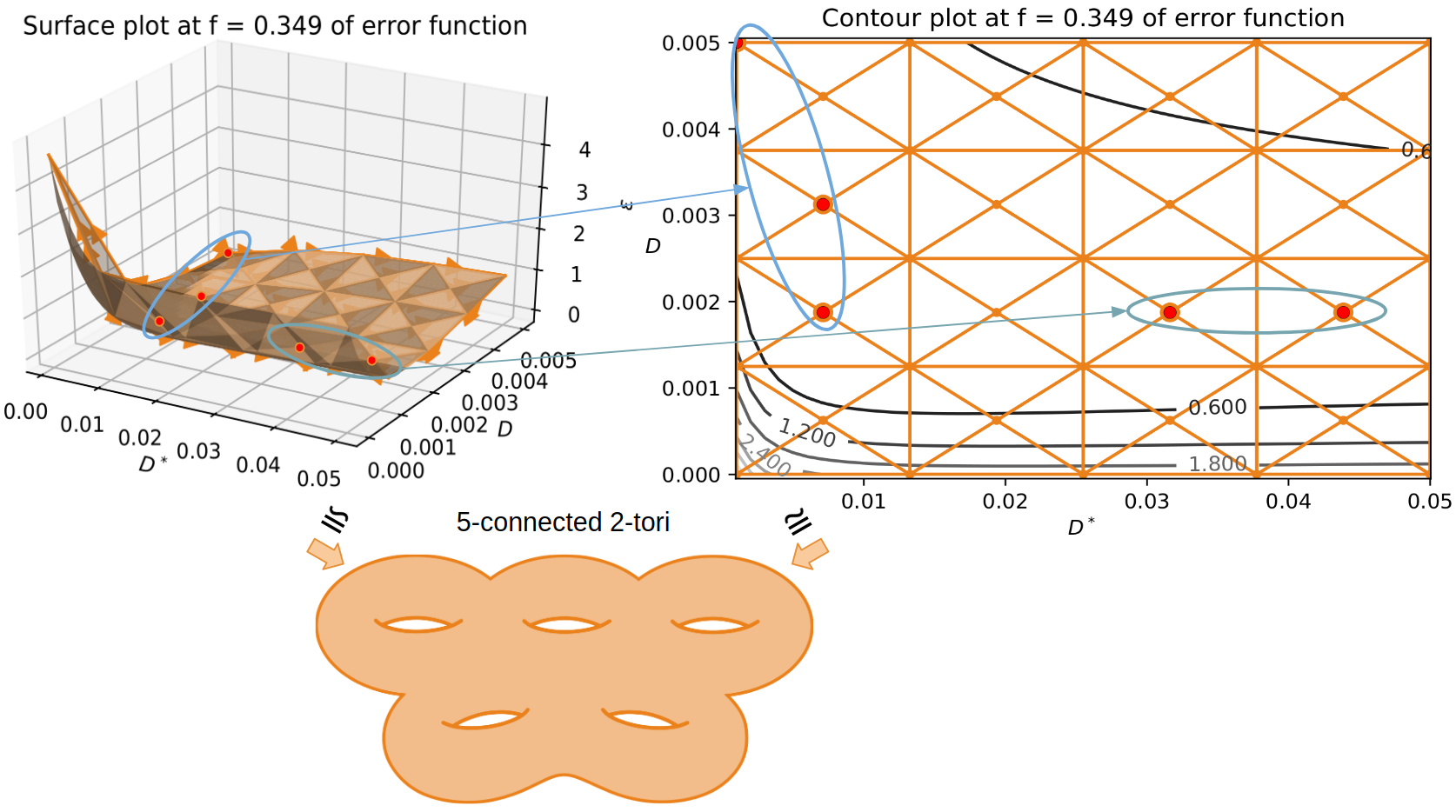

Supplement: Supplementary file 2 [file Data_Sheet_2.ZIP › topopro-master/figs/bimodal_sh.png]

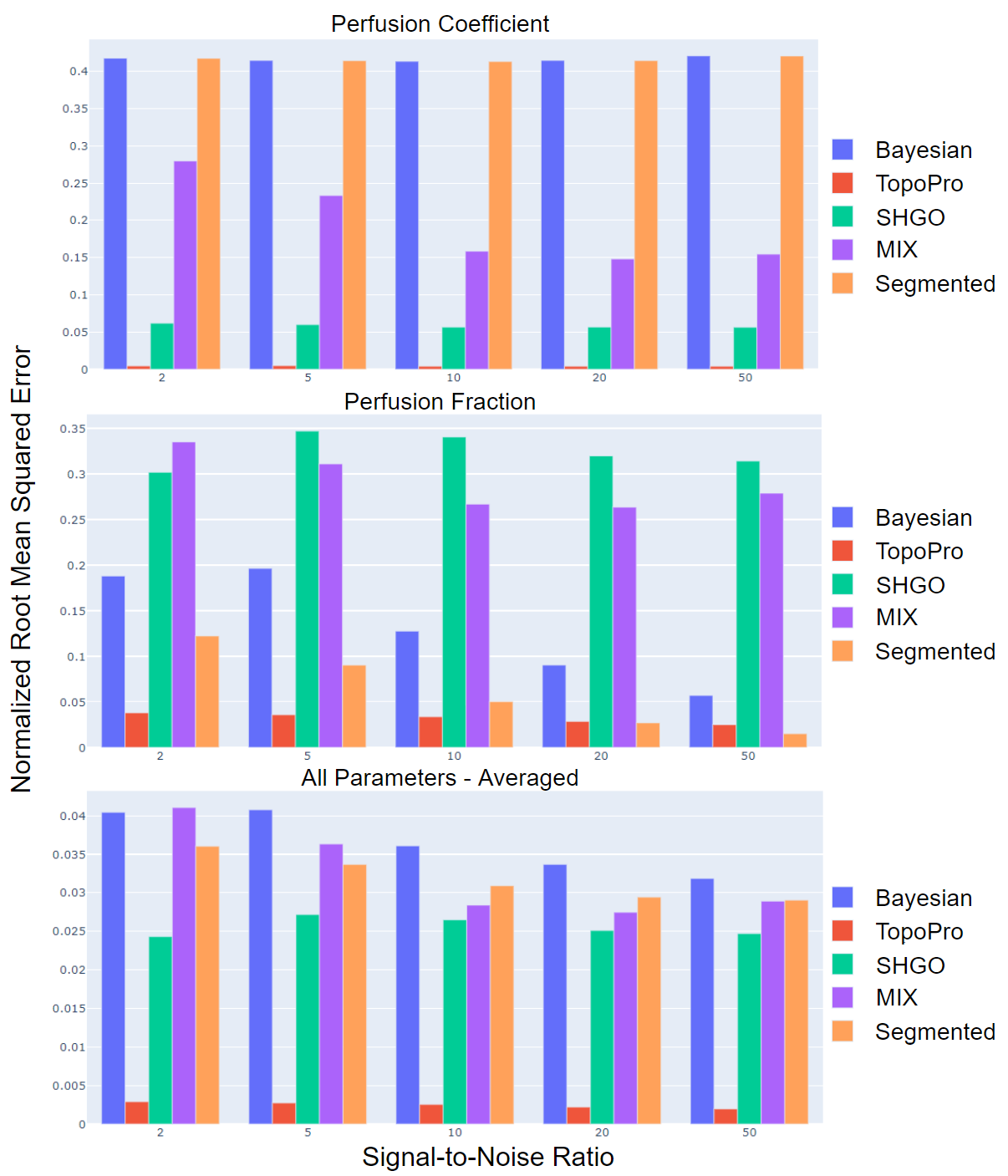

Supplement: Supplementary file 2 [file Data_Sheet_2.ZIP › topopro-master/figs/comparison_bar_chart.PNG]

Contour plot at  $f = 0.0$  of error function

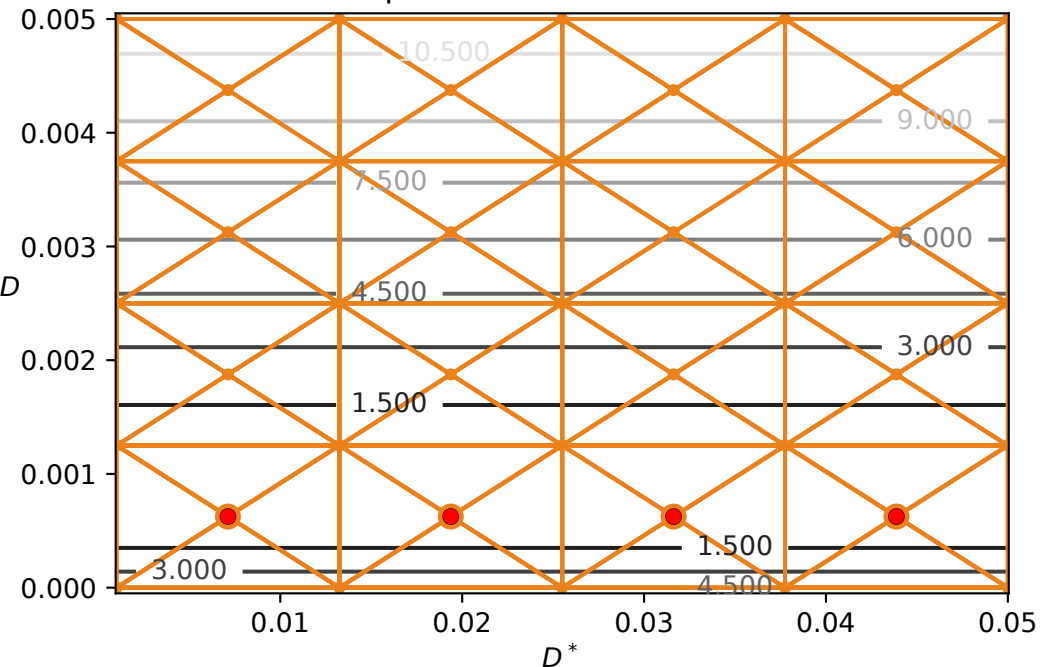

Surface plot at  $f = 0.0$  of error function

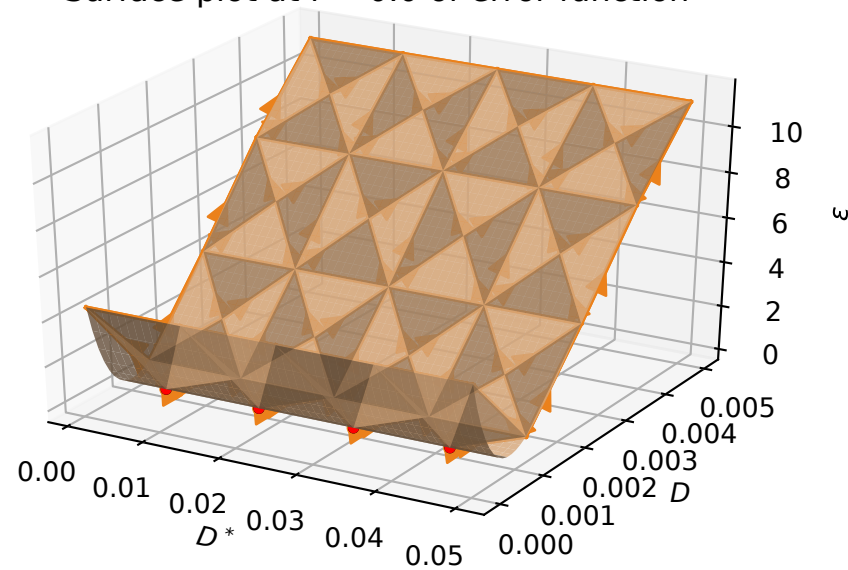

Supplement: Supplementary file 2 [file Data_Sheet_2.ZIP › topopro-master/figs/cont_surf_f_0.pdf]

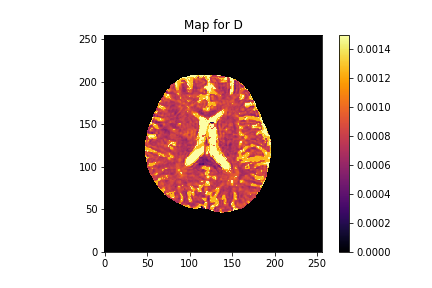

Supplement: Supplementary file 2 [file Data_Sheet_2.ZIP › topopro-master/figs/diffusion_coeff.png]

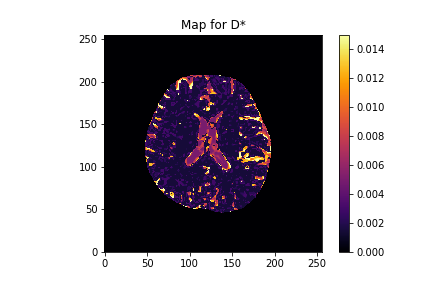

Supplement: Supplementary file 2 [file Data_Sheet_2.ZIP › topopro-master/figs/perfusion_coeff.png]

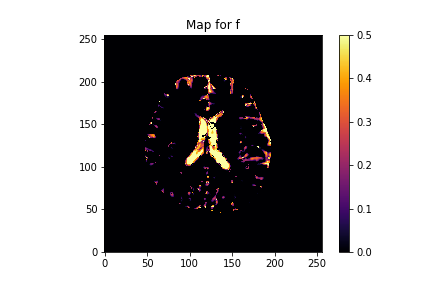

Supplement: Supplementary file 2 [file Data_Sheet_2.ZIP › topopro-master/figs/perfusion_fraction.png]

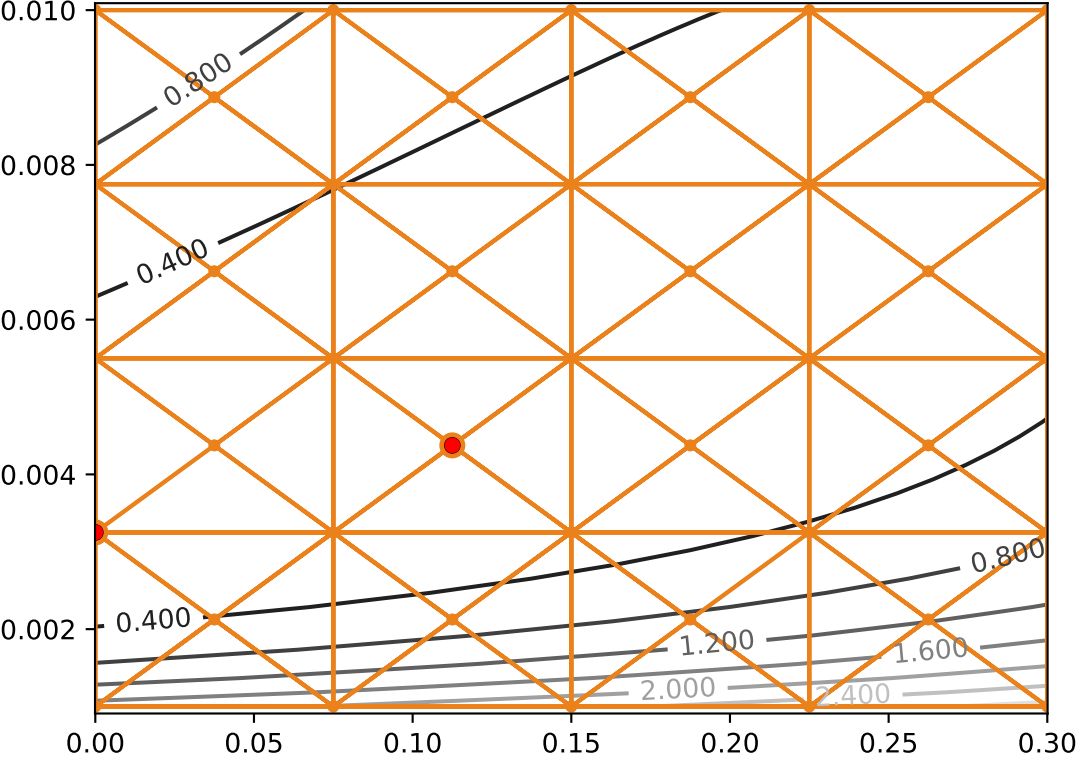

Supplement: Supplementary file 2 [file Data_Sheet_2.ZIP › topopro-master/notebooks/fig/complex.pdf]
